# Geographical disparities in treatment and health care costs for end-of-life cancer patients in China: a retrospective study

**DOI:** 10.1186/s12885-018-5237-1

**Published:** 2019-01-08

**Authors:** Anli Leng, Jun Jing, Stephen Nicholas, Jian Wang

**Affiliations:** 1 Center for Health Economics Experiment and Public Policy, School of Public Health, Shandong University, No. 44 Wenhuaxi Road, Lixia District, Jinan, 250012 China; 2 Key Laboratory of Health Economics and Policy Research NHFPC, Shandong University, Jinan, China; 3 Research Center for Public Health, Tsinghua University, Room B408, Medical School, Beijing, 100084 China; 4 School of Economics and School of Management, Tianjin Normal University, West Bin Shui Avenue, Tianjin, 300074 China; 5 TOP Education Institute, 1 Central Avenue, Australian Technology Park, Eveleigh, Sydney, NSW 2015 Australia; 6 Newcastle Business School, University of Newcastle, University Drive, Newcastle, NSW Australia; 7 Dong Furen Institute of Economic and Social Development, Wuhan University, 54 Dongsi Lishi Hutong, Beijing, 100010 China

**Keywords:** Geographic disparities, Cancer treatments, Health care costs, End-of-life (EOL)

## Abstract

**Background:**

Cancer imposes substantial burdens on cancer suffers, their families and the health system, especially in the end of life (EOL) of care patients. There are few developing country studies of EOL health care costs and no specialist studies of the disparities in cancer treatment and care costs by geographical location in China. We sought to examine geographical disparities in the types of cancer treatments and care costs during the last 3 months of life for Chinese cancer patients.

**Methods:**

Using snowball sampling and face-to-face interviews, field research was conducted with a specialist questionnaire. Data were collected on 792 cancer patients who died between July 2013 and June 2016 in China. Total EOL health care costs were modeled using generalized linear models (GLMs) with log link and gamma distribution.

**Results:**

Total health care costs were highest for urban (US$12,501) and western region (US$9808) patients and lowest for rural (US$5996) and central region (US$5814) patients. Our study revealed about 40% of the health care expenses occur in the last three months of life, and was mainly driven by hospital costs that accounted for about 70% of EOL expenditures. Patients faced out-of-pocket expenses for health care, with the ability to borrow from family and friends also impacting the type of treatment and health facility. Life-extending treatments per cancer patient was about two times that of patients receiving conservative treatments.Urban patients were more likely to receive life-extending treatments, financed by higher incomes and a greater capacity to borrow from family and friends to bridge the gap between health insurance reimbursements and out-of-pocket expenditures. Cancer patients in western region and urban area were significantly more likely to access hospice care.

**Conclusions:**

We found significant urban-rural and regional disparities in EOL types of cancer treatment, utilization of medical care and the health care expenditures. The EOL cancer care costs imposed heavy economic burdens in China.We recommend better clinical guidelines, improved EOL conversations and fuller information on treatment regimes among patients, family caregivers and doctors. Policies and information should pay more attention to palliative care options and the socio-cultural context of cancer care decision-making by family.

**Electronic supplementary material:**

The online version of this article (10.1186/s12885-018-5237-1) contains supplementary material, which is available to authorized users.

## Background

Cancer is the second leading cause of death globally, accounted for 8.8 million deaths in 2015 or nearly 1 in 6 of all global deaths [1]. Adjusting for country-specific changes in population growth and population ageing, GLOBOCAN estimated that global cancer deaths would rise 72% from 7.6 million in 2008 to 13.2 million deaths by 2030 [2]. Cancer is the leading cause of death in China, with the mortality rate for urban residents 164.35/100 thousand and 154.98/100 thousand for rural residents [3]. Population aging and growth saw the number of cancer deaths increase by 73.8% during ten years from 2000 to 2011 [4].

Cancer imposes a substantial economic burden on cancer suffers, their families and the health system. The global annual costs of cancer were estimated to be US$1.16 trillion in 2010 [1]. In the United States, direct medical costs for cancer care was US$77.4 billion in 2008 [5], increasing to US$124.57 billion in 2010 [6]. In England, colorectal, breast, prostate, and lung cancer cost were estimated to be US$2.34 billion annually for hospital care alone in 2010 [7]. For the European Union (EU), cancer costs were estimated to be €51 billion in 2009, with 40% accounted for by direct health care costs [8]. Typically, the cancer care cost curve has a distinctive U-shape distribution, with the most resource-intensive stage of health care at the end-of-life (EOL) [7, 9–11]. Previous studies in United States estimated that one third of all direct medical costs of cancer treatment occur in the final year of the disease, with approximately 80% of the final year amount spent in the last month of care [12]. Compared with non-cancer patients, cancer patients incur substantially higher EOL costs [13].

Previous research on EOL medical costs has focused on developed countries, such as United States [13–18], England [19–23], Australia [10, 24] and Canada [16, 25], with few studies of EOL medical costs in emerging and developing countries. Also, previous research on the medical costs of EOL care identified socioeconomic disparities, such as racial [15] and geographic disparities [14, 15], and different EOL treatment regimes [26], including inpatient care [10, 15, 17, 20, 23], outpatient care [14, 26] and hospice care [16, 22, 25]. Previous studies also found that patients’ age [24], place of residence [14], nationality [15] and treatment [27] were associated with end-of-life resource use and costs. Besides a developed country bias, previous EOL cancer cost studies have frequently used data not collected to answer specific EOL cost questions [20] and heavily relied on publicly available data sets [16, 19, 25–29]. We address both these short-comings, utilizing a specific EOL cancer survey for industrializing China.

For developing countries, China provides an important case study of EOL medical costs for cancer care. Besides being the largest developing country in the world, China presents a significant dual urban-rural economic structure [30] and also disparities in regional economic structure, with a persistent income gap between urban and rural residents and industrialized versus agricultural provinces. There is a large urban-rural and regional medical consumption gap reflected in a suppressed demand for medical consumption in rural areas, with per capita health cost in rural areas about half of that in urban areas [31]. Not surprisingly, there are large inequalities between rural and urban residents and residents in poor versus rich provinces in health care level, access and utilization of health services, health financing and the utilization of health resources [32, 33]. These urban-rural and regional disparities impact the cost, access and utilization of EOL health services [34–36].

In spite of these rural-urban and regional disparities in medical expenditures and access and utilization of health services, previous Chinese studies have not specifically examined the rural-urban and regional differences in the costs of EOL care for patients with cancer. This paper examines geographical disparities in cancer health care costs during the last 3 months of life for deceased cancer patients who died between July 2013 and June 2016 in China.

## Methods

### Data sources

Between July 1 and August 312,016, we conducted a retrospective study of caregivers of deceased cancer patients, who died from cancer in China between June 2013 and June 2016. There is no consistent definition of end-of-life period in cancer care, where EOL can refer to the last 6, 3 or 1 month, as well as the last 14 days, of life [37]. EOL in our study is defined as the last three months of life.

Snowball sampling (SS) is an established method to obtain samples from hard-to-reach human populations through chain referrals [38, 39, 57]. Widely used to estimate health related problems, the snowball sampling method recruits research samples through social contacts in the target population’s social network [39, 40, 57]. Our hidden population is reflected both in the reluctance of families to publicly acknowledge their relatives’ cancer and the privacy problems that exclude accessing patients’ hospital and insurance records. Even if they were accessible, such records do not include the full range of expenditures incurred by families with relatives with cancer.

But, snowball sampling poses problems of representativeness, since respondents are not randomly drawn, but are dependent on the subjective choices of the respondents first accessed, which may pose problems of selection bias limiting the claims to generality [58, 59]. To address this problem, we undertook a rigorous recruitment chain process that began with “seeds” of the target population. Through health care facilities, twenty professionally field research trained medical students established contact with relatives, village doctors, community doctors and caregivers, who had personal or professional contact with the deceased patients. This resulted in initial interviews. To reduce any bias in the selected socio-demographic characteristics, seeds were selected based on the demographic characteristics of the population-based cancer deaths in China. The seeds were selected to reflect urban and rural location and from different socio-economic backgrounds, marital status and ages. All seeds met two inclusion criteria. First, they had cancer according to the International Statistical Classification of Diseases and Related Health Problems, Tenth Revision (ICD-10), cancer diagnosis codes. Second, cancer patients died between June 2013 and June 2016. All surveys were conducted face-to-face, with participants provided the option of receiving a 25RMB cash gift. Each respondent was given two coupons to recruit other caregivers using a tracked coupon system until an equilibrium sample size was achieved on key variables. This method allowed the research team to identify the social network structure while allowing participants to remain anonymous if desired. Data collection continued for two months, with a maximum chain length of six waves. Selection bias was also partially addressed by collecting a large sample. Importantly, we found no statistically significant differences in socio-demographic characteristics between our sample and population-based cancer deaths in China [4, 41].

Completed by the deceased patient’s caregiver, the interview questionnaire consisted of the deceased patient’s socioeconomic characteristics, treatments decisions (surgery, radiotherapy, chemotherapy, targeted cancer therapy and hospice), health care utilization (hospitalization and outpatient), health care cost (total health care expenditure, the out-of-pocket payment, reimbursement amount), and borrowing money (the amount of money borrowed and the days to repay) from diagnosis of cancer to death, with particular emphasis on the last three months of their life. The self-made questionnaire was presented in details in Additional file 1. The interviews provided a retrospective database, including health service expenses related to the patient’s cancer treatment and related health cost issues [27, 42].

### Study population

**Fig. 1:**
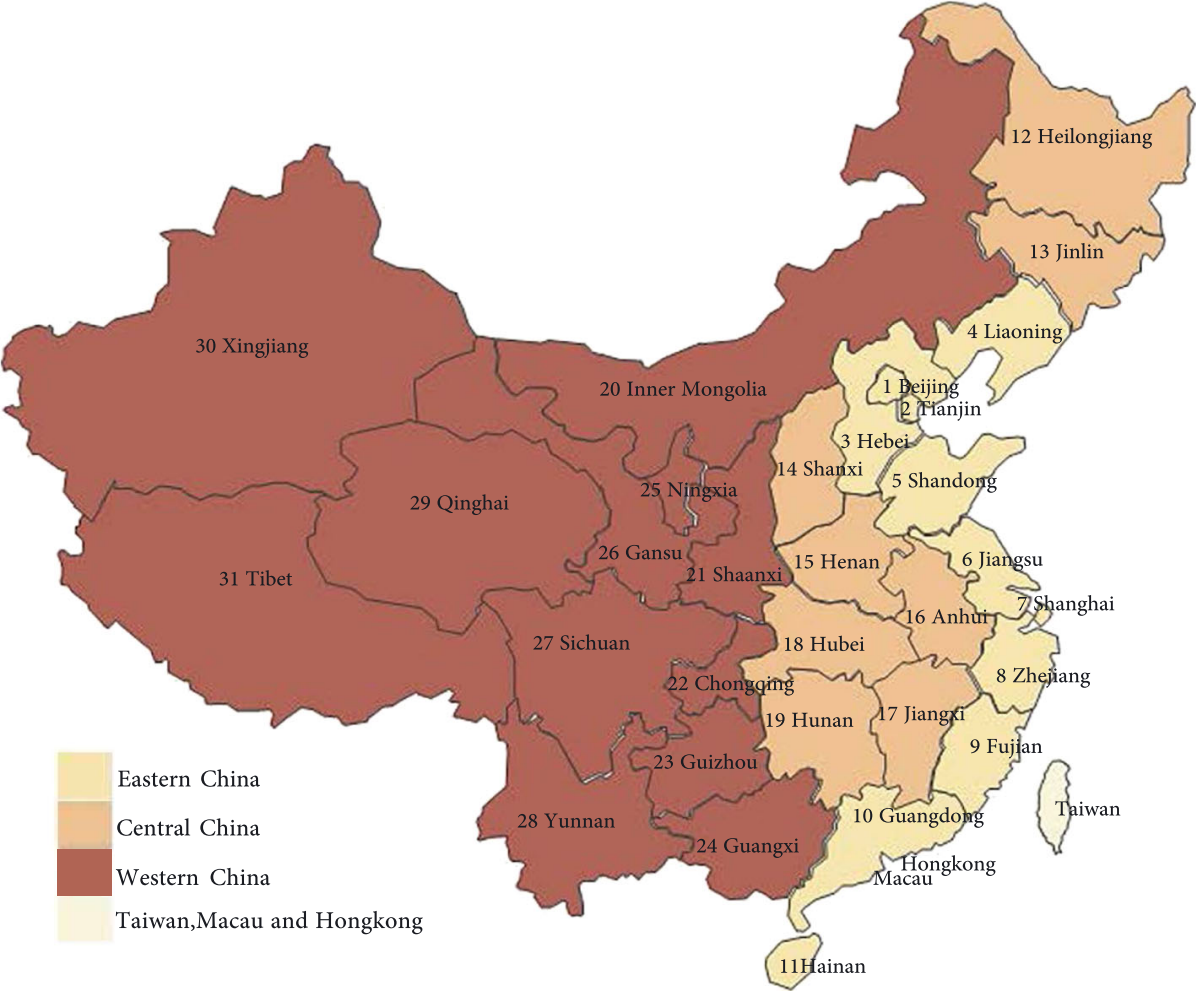
Geographical divisions in China. Legend: Eastern China (including:1 Beijing, 2 Tianjin, 3 Hebei, 4 Liaoning, 5 Shandong, 6 Jiangsu, 7 Shanghai,8 Zhejiang, 9 Fujian, 10 Guangdong, and 11 Hainan). Central China (including:12 Heilongjiang,13 Jilin, 14 Shanxi,15 Henan,16 Anhui,17 Jiangxi, 18 Hubei, and 19 Hunan). Western China (including: 20 Inner Mongolia, 21 Shan’xi, 22 Chongqing, 23 Guizhou, 24 Guangxi, 25 Ningxia, 26 Gansu, 27 Sichuan, 28 Yunnan, 29 Qinghai, 30 Xinjiang, and 31 Tibet). Taiwan, Macau and Hongkong

Deceased patients were included in the study if they met two inclusion criteria. First, they had cancer according to the International Statistical Classification of Diseases and Related Health Problems, Tenth Revision (ICD-10), cancer diagnosis codes, which the field researchers confirmed using the ICD-10 classification in face-to-face interviews with carers. Second, cancer patients died between June 2013 and June 2016. Our data include health care costs from diagnosis of cancer to their death, with specific analysis on EOL (or last three months of life) health care costs. Cancer health care costs comprised both hospital (inpatient) costs and outpatient costs. A small number of patients with no detailed medical costs were excluded. In total, 829 caregivers were included in our research; 62 caregivers (6.96%) refused to participate, giving us a 93% response rate. Due to some missing data,the sample consisted of 792 caregivers with advanced cancer patients from across China, with Shandong Province (47.6%), Shan’xi (11.24%), Yunnan (9.47%), Jiangxi 7.83%), Henan (5.30%), Anhui (4.92) accounting for the bulk of the respondents and 13.64% from other provinces, autonomous regions and municipalities. As shown in Fig. 1, we grouped the provinces into western (Inner Mongolia, Guangxi, Chongqing, Sichuan, Guizhou, Yunnan, Tibet, Shan’xi, Gansu, Qinghai, Ningxia, Xinjiang), central (Heilongjiang, Jilin, Shanxi, Anhui, Jiangxi, Henan, Hubei, and Hunan) and eastern (Beijing, Tianjin, Shanghai, Hebei, Liaoning, Jiangsu, Zhejiang, Fujian, Shandong, Guangdong, and Hainan) regions (Please see Fig. 1). One hundred and ninety five (195) patients were from urban areas and 597 patients from rural areas. By type of cancer, the sample comprised 182 lung, 128 gastric, 132 liver, 89 esophagus cancer patients, 50 intestinal cancer sufferers, and 211 “other” types of cancer patients.

### Outcome measures

The outcome measure consisted of two periods of health care expenses. One was total cancer care costs between diagnosis with cancer and death. The second was EOL health care costs incurred in the last 3 months of life. The structure of medical costs in our study consisted of three parts: the cancer health care expenditure, the out-of-pocket payments and the reimbursement costs. Cancer health care costs included life-extending and conservative hospital and outpatient treatments, such as surgery, radiotherapy, chemotherapy and targeted therapy, drug therapy, Chinese acupuncture and traditional medical treatments. We requested the participants to recall the health care costs of each treatment carefully. In cases when caregivers were uncertain, information was sought from the patient’s family doctor. When available, patients’ medical records were used to recall some of the medical costs and expense invoices were used to calculate the amounts. Importantly, the main caregivers remembered the amount of total health care costs, the reimbursement costs and the out-of-pocket payments clearly, because it was a large family expense and because the reimbursement procedures were complex, with patients and their family paying medical expenses before seeking reimbursement from their medical insurance. All costs were adjusted to 2016 prices and based on a currency exchange rate of the 6.6423 yuan to US$1 in 2016.

### Analysis

Descriptive statistics were performed to compare the distribution of patient social-demographic characteristics and the types of cancer treatment across geographic locations. Statistical comparisons were performed using Pearson’s chi-squared test and Student’s t-test, with differences between groups by geographical location assessed through analysis of variance (ANOVA). Previously used in end-of-life cost analyses [14, 15, 19], generalized linear models (GLMs), with log link and gamma distribution, were used to model total EOL health care costs [43]. A small number of cancer patients who received no hospital treatment were included and allocated zero costs. All analyses were conducted using STATA, version 14 and significance was assessed at level of 0.05.

## Results

### Social-demographic characteristics by urban-rural location and the three regions

For our sample of 792 patients, Table 1 presents the distribution of social-demographic characteristics by urban-rural location and the three regions. There were no significant urban-rural differences by marital status (roughly 80% married) and age (about 64 years old). Urban-rural differences were evident in a range of social-demographic characteristics: compared with the patients in rural areas, patients in urban areas were less likely to be male; more likely to have steady work and to earn higher incomes and to have longer survival time from diagnosis to death. As expected, there were significant differences in the occupational mix, with rural areas having more farmers and urban areas more retired patients. For urban patients, the nearest medical institution was municipal level or above hospitals (56.41%), while rural patients overwhelmingly had access to village clinics (47.4%). While urban cancer patients had a slightly longer travel distances to the nearest medical institution, the quality of urban medical services in the form of municipal and higher hospitals was higher than health facilities in rural areas.

**Table 1:** Characteristics of the study sample by geographical location (*N* = 792)

Characteristics	Urban (*n* = 195)			Rural (*n* = 597)			*p*- value	Western (*n* = 162)			Central (*n* = 242)			Eastern (*n* = 388)			*p* - value
	n	%	mean (SD)	n	%	mean (SD)		n	%	mean (SD)	n	%	mean (SD)	n	%	Mean (SD)	
Gender			NA			NA	0.00			NA			NA			NA	0.35
Male	116	59.49		423	70.85			110	67.90		173	71.49		256	65.98		
Female	79	40.51		174	29.15			52	32.10		69	28.51		132	34.02		
Age			64.75 (14.42)			64.01 (12.78)	0.49			61.15 (12.86)			64.29 (13.35)			65.42 (13.06)	0.01
Age < 45	15	7.69		44	7.37			20	12.35		18	7.44		21	5.41		
Age 45–54	37	18.97		77	12.90			28	17.28		33	13.64		53	13.66		
Age 55–64	40	20.51		174	29.15			53	32.72		65	26.86		96	24.74		
Age 65–74	48	24.62		169	28.31			36	22.22		71	29.34		110	28.35		
Age 75-	55	28.21		133	22.28			25	15.43		55	22.70		108	27.84		
Marriages			NA			NA	0.08			NA			NA			NA	0.02
Married	156	80		501	83.92			144	88.89		204	84.30		309	79.64		
Other (unmarried/widowed/divorced)	39	20		96	16.08			18	11.11		38	15.70		79	20.36		
Nation			NA			NA	0.22			NA			NA			NA	
Han	188	96.41		562	94.14			125	77.16		240	99.17		385	99.20		0.00
Minority	7	3.59		35	5.86			37	22.84		2	0.83		3	0.77		
Residence			NA			NA	NA			NA			NA			NA	0.00
Urban	NA	NA		NA	NA			47	29.01		28	11.57		120	30.93		
Rural	NA	NA		NA	NA			115	70.99		214	88.43		268	69.07		
Occupation			NA			NA	0.00			NA			NA			NA	0.00
Farmer	37	18.97		476	79.73			106	65.43		178	73.55		229	59.02		
Steady workers	27	13.85		42	7.04			12	7.41		21	8.68		36	9.28		
Non-regular workers	55	28.21		55	9.21			19	11.73		28	11.57		63	16.24		
The retired	76	38.97		24	4.02			25	15.43		15	6.20		60	15.46		
Annual per-capita income ($)^a^			28.05 (31.29)			10.78 (14.06)	0.00			16.91 (17.50)			8.90 (17.13)			18.07 (23.67)	0.00
Low income level (≤ $421)	14	7.18		165	27.64			15	9.26		83	34.30		81	20.88		
Below average level($422–$1505)	42	21.54		283	47.40			76	46.91		117	48.35		132	34.02		
Average level ($1506–$4015)	61	31.28		80	13.40			40	24.69		24	9.92		77	19.85		
Above average level (≥$4016)	78	40.00		69	11.56			31	19.14		18	7.43		98	25.26		
The nearest medical institutions			NA			NA	0.00			NA			NA			NA	0.00
Village clinics	1	0.51		283	47.40			58	35.80		67	27.69		159	40.98		
Town health center	5	2.56		113	18.93			11	6.79		67	27.69		40	10.31		
County hospital	53	27.18		120	20.10			55	33.95		33	13.64		85	21.91		
Municipal level or above hospitals	110	56.41		5	0.84			28	17.28		14	5.79		73	18.81		
Private clinic	1	0.51		57	9.55			0	0.00		56	23.14		2	0.52		
Other	25	12.82		19	3.18			0	0.00		5	2.07		0	0.00		
Distance to the nearest medical institutions			2.77 (2.58)			2.70 (4.19)	0.83			3.62 (5.86)			3.10 (3.77)			2.11 (2.50)	0.00
< 1 km	29	14.87		195	32.66			37	22.84		44	18.18		143	36.86		
1-5 km	137	70.26		301	50.42			93	57.41		146	60.33		199	51.29		
>5 km	29	14.87		100	16.92			32	19.75		52	21.49		46	11.85		

^a^Based on a currency exchange rate of the 6.6423 yuan to US$1.00 in 2016

From Table 1, patients in the eastern region were older, and those in the west region younger, which helps explain the higher percentage of widowers in the eastern group and married patients in the western region. There were fewer farmers and more non-regular workers in the eastern group, with a higher income than those in the central region. Western region patients in our study were more likely to be minorities, married and urban, with fewer in the low-income category and more accessing country hospitals than the other regions. Eastern province cancer patients were most likely to be urban and use village clinics, while central cancer patients were more likely to use private clinics.The eastern group patients were located the closest to a health facility. In the central region, there were fewer urban patients (11.57%) and more rural patients (88.43%) than the other regions.

### Days from being diagnosed with cancer to dying and total cancer costs by urban-rural location and the three regions

Table 2 reports that urban cancer patients had longer survival time from diagnosis to death (549 days) than rural patients (448 days). Whether urban or rural, about one fifth of patients died within three months after they were diagnosed with cancer. Compared with rural patients, urban patients’ total cancer care costs, out-of-pocket expenses and reimbursement, were more than two times those of rural patients. Also,the total cancer care costs as well as out-of-pocket expenses were highest in the eastern region.The cost distributions were presented in details in Additional file 2.

**Table 2:** Days from being diagnosed with cancer to dying and total cancer costs (*N* = 792)

Characteristics	Urban (N = 195)		Rural (N = 597)		*P* - value	Western (*N* = 162)		Central (*N* = 242)		Eastern (*N* = 388)		*P* - value
	N (%)	Mean (SD)	N (%)	Mean (SD)		N (%)	Mean (SD)	N (%)	Mean (SD)	N (%)	Mean (SD)	
Days from being diagnosed with cancer to dying		549 (799)		448 (487)	0.02		576 (696)		451 (536)		444 (550)	0.00
≤3 months	35 (17.95)		96 (16.08)			30 (18.52)		43 (17.77)		58 (14.95)		
3 months–6 months	33 (16.92)		123 (20.60)			35 (21.60)		58 (23.97)		63 (16.24)		
6 months-1 year	57 (29.23)		157 (26.30)			29 (17.90)		54 (22.31)		131 (33.76)		
1 year-2 years	40 (20.51)		137 (22.95)			37 (22.84)		50 (20.66)		90 (23.20)		
≥ 2 years	30 (15.38)		84 (14.07)			31 (19.14)		37 (15.29)		46 (11.86)		
Per capita expenditures, US$		32,671 (45,576)		15,541 (15,955)	0.00		19,791 (18,132)		16,579 (20,423)		21,703 (33,747)	0.03
Including:out-of-pocket expenses		17,051 (23,731)		9405 (10,623)	0.00		8979 (11,036)		10,940 (15,516)		12,483 (16,579)	0.05
Reimbursements		15,620 (29,698)		6136 (8202)	0.00		10,812 (12,365)		5639 (8642)		9220 (21,479)	0.00

US$ Based on a currency exchange rate of the 6.6423 yuan to US$1.00 in 2016.Median and inter-quartile range cost date are available form the authors

### Types of cancer care in the last three months by geographical location

Table 3 reports that there are significant differences by geographical location in terms of treatment decision and health care utilization. Approximately 64% of urban end-stage cancer patients received aggressive life-extending treatments, such as surgery, chemotherapy and radiotherapy, in urban areas, compared to only 42% of rural cancer patients. Consistently, the proportion hospitalized, the per capita hospital admissions and and per capita hospital days for urban patients were higher than for rural patients. Rural patients were more likely to receive conservative treatments, such as drug therapy and traditional Chinese medicine, or no treatment. Although relatively higher in urban and western areas, the proportion of patients receiving palliative care was very low in China, ranging range from 2.68 to 12.31%. We found the average per capita hospital admissions and per capita hospital days were highest in the eastern region.

**Table 3:** Types of cancer care in the last three months by geographical location (*N* = 792)

Characteristics	Urban (*N* = 195)	Rural (*N* = 597)	*P-* value	Western (*N* = 162)	Central (*N* = 242)	Eastern (*N* = 388)	*P* - value
Type of treatment (before doctors determined no further treatment to be provided), No. (%)			0.00				0.00
Life-extending treatment	124 (63.59)	255 (42.71)		90 (55.56)	92 (38.02)	197 (50.77)	
Conservative treatment	71 (36.41)	317 (57.29)		72(44.44)	150(61.98)	191 (49.23)	
Health care utilization							
Hospital (include hospice), No. (%)			0.00				0.00
Yes	179 (91.79)	431 (72.19)		139 (85.80)	172 (71.07)	299 (77.06)	
No	16 (8.21)	166 (27.81)		23 (14.20)	70 (28.94)	89 (22.94)	
Per capita hospital admissions, mean (SD)	2.13 (2.02)	1.51 (1.62)	0.00	2.02 (1.52)	1.44 (1.43)	1.66 (1.97)	0.00
Per capita hospital days, mean (SD)	41.13 (30.00)	23.49 (25.28)		27.22 (22.23)	25.24 (26.77)	29.72 (29.90)	0.00
Hospice care, No. (%)			0.00				0.00
Yes	24 (12.31)	16 (2.68)		18 (11.11)	10 (4.13)	12 (3.09)	
No	171 (87.69)	538 (97.32)		98 (88.89)	232 (95.87)	376 (96.81)	

### Health care costs at the EOL by geographical location and types of treatment

Table 4 presents the mean EOL health care costs by geographical location and types of cancer treatment. The mean total care cost for urban cancer patients was about $12,501, almost twice that of in rural areas ($5996). Stratified by types of cancer treatment, we found that the patients who received the life-extending treatment spent about twice the amount on health care costs compared to patients with conservative treatments ($10,601 > $4841). Across regions, the total expenses were higher in the western region ($9808) than in the central ($5814) or eastern ($7756) regions. Although basic medical insurance coverage is nearly universal in China, which reduced some economic burden of patients, patients still need to pay about half their health care costs (out-of-pocket expenses). Hospitalization was the most important component of total care costs, ranging between 62 and 78% of total health care costs. The mean total costs of hospitalization in the last three months of life for urban patients was $10,085, compared to $4320 for rural cancer patients. The mean out-of-pocket hospitalization costs accounted for approximately half of the total hospitalization costs. The detailed cost distributions were in Additional file 3.

**Table 4:** Health care costs of the EOL patients by geographical location and type of treatment (*N* = 792)

Characteristics	Urban (*N* = 195)	Rural (*N* = 597)	*P -* value	Life-extending treatment (*N* = 379)	Conservative treatment (*N* = 413)	P- value	Western (*N* = 162)	Central (*N* = 242)	Eastern (*N* = 388)	*P* - value
Health expenditures										
Per capita expenditures, mean (SD), US$	12,501 (15,711)	5996 (6359)	0.00	10,601 (12,611)	4841 (5550)	0.00	9808 (8.910)	5814 (6372)	7756 (11,948)	0.00
Including:out-of-pocket	6225 (7221)	3893 (4693)	0.00	5852 (6574)	3196 (3914)	0.00	5063 (5917)	3976 (4656)	4516 (5802)	0.00
reimbursement	6276 (11,558)	2103 (2972)	0.00	4749 (8768)	1645 (2596)	0.00	4745 (5301)	1838 (2772)	3240 (8270)	0.00
Inpatient health care utilization										
Hospitalization in hospital, No. (%)	179 (91.79)	431 (72.19)	0.00	379 (100)	274 (66.34)	0.00	142 (87.65)	169 (69.83)	299 (77.06)	0.00
Per capita hospital expenditures, mean (SD), US$	10,085 (12,384)	4320 (5798)	0.00	8467 (10,397)	3236 (4516)	0.00	8225 (8637)	4086 (5740)	5700 (9223)	0.00
Including:out-of-pocket	4668 (5696)	2570 (4241)	0.00	4409 (5738)	1873 (3094)	0.00	3978 (5773)	2490 (4045)	3075 (4598)	0.00
reimbursement	5417 (9025)	1750 (2650)	0.00	4058 (6977)	1363 (2275)	0.00	4247 (5127)	1596 (2489)	2625 (6327)	0.00
Hospital costs % total health care costs	74.23	67.54	0.00	75.45	61.93	0.00	78.87	65.69	68.05	0.00

US$ Based on a currency exchange rate of the 6.6423 yuan to US$1.00 in 2016.Median and inter-quartile range cost date are available from the authors

**Fig. 2:**
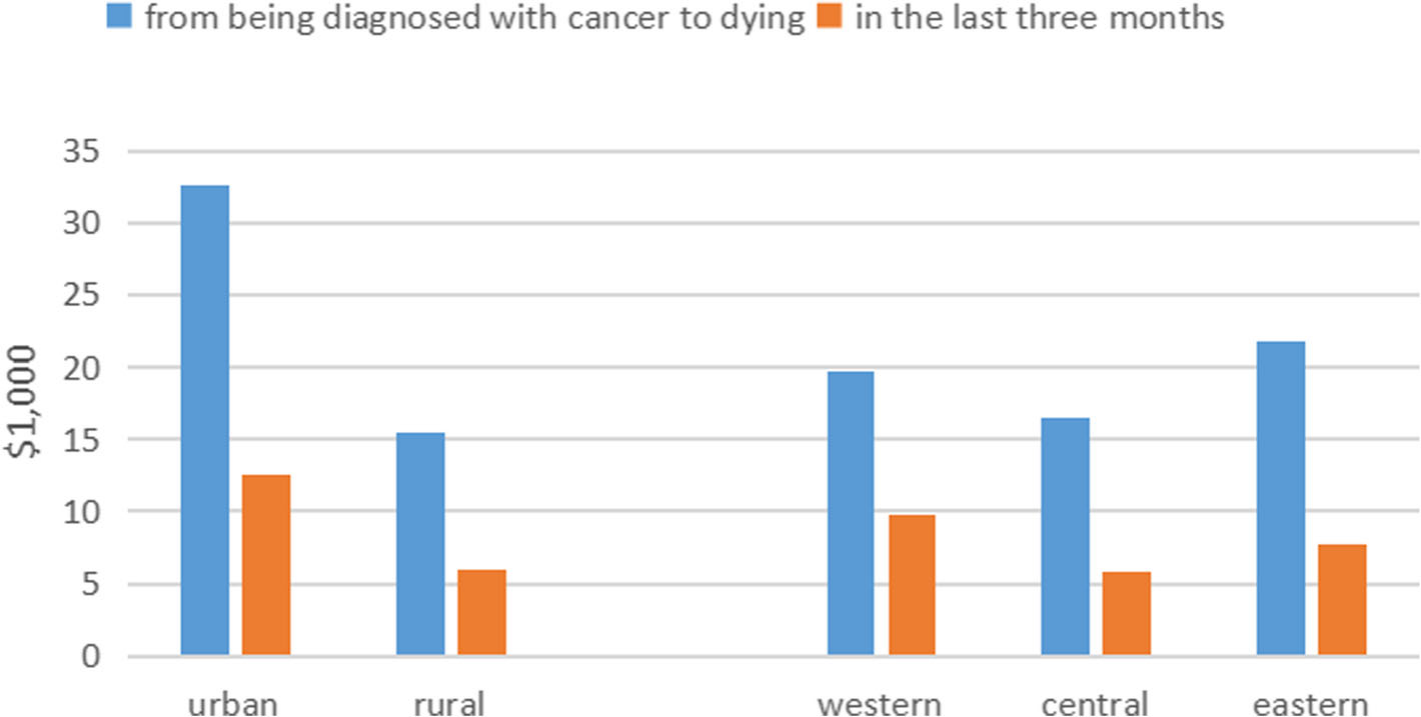
EOL care costs as proportion of entire cancer period costs by geographical location. Legend: from being diagnosed with cancer to dying in the last three months

Figure 2 shows that the care costs in the last three months accounted for roughly 40% of the total cancer expenses for urban and rural patients and those from eastern and central regions. The proportion of health care costs in the EOL patients in western zone was 52% of total health care costs, due to their higher hospital costs.

### Generalized linear model for EOL health care costs

Table 5 presents the model-based cost estimates using a generalized linear model. Urban-rural location, types of cancer treatment and whether hospitalized or not were the significant factors explaining EOL health care costs. After adjusting for other social-demographic and cancer factors, the mean health care cost in the last three months of life was significantly higher in urban areas compared to rural areas. As expected, patients receiving life-extending treatment also had significantly higher costs than those received conservative. There were no significant differences in EOL care costs by regions in Table 5.

**Table 5:** Generalized linear model for total EOL health care costs by types of cancer care and geographical location (*N* = 792)

Characteristics	Adjusted cost Ratio (95% CI)^ab^	*P* - value
Urban-rural location		
Rural	1	
Urban	1.58 (1.32–1.91)	0.000
Types of cancer treatment		
Conservative treatment	1	
Life-extending treatment	1.46 (1.28–1.69)	0.000
Utilization of medical care		
Hospitalization (no vs. yes)	2.70 (2.28–3.19)	0.000
Hospice care (no/not sure vs. yes)	1.01 (0.80–1.27)	0.943
Regions		
Western	1	
Central	0.86 (0.68–1.07)	0.171
Eastern	0.85 (0.69–1.05)	0.129

^a^Adjusted for age, gender, nation, personal income, marital status, occupation, cancer types, days from being diagnosed to dying from cancer, the type of nearest medical facility and the distance to the nearest medical facility

^b^Cost ratio is calculated as exp. (estimate), which represents the cost ratio in RMB value between the specified group and the reference group

### Borrowing to meet EOL health care costs

EOL health care costs imposed significant economic burdens on cancer patients and their families. It was common for cancer patients to borrow from their relatives and friends to finance cancer health care costs, with 260 or 32.83% of patients borrowing from relatives and friends. Compared to rural cancer patients, Table 6 shows that urban cancer patients borrowed more money for cancer health costs, but required less time to repay the loan, than rural cancer patients. The mean borrowing of patients received life-extending treatment was about twice that of those receiving conservative treatments. Table 6 also shows that borrowing was highest in the central zone, with a longer pay back time than other regions, while western patients borrowed least and eastern patients had the shortest pay-back time.

**Table 6:** Borrowing to meet EOL health care costs (*N* = 260)

Characteristics	Amounts (US$)	*P-* value	Repayment (years)	*P-* value
	Mean (SD)		Mean (SD)	
Urban-rural location		0.11		0.86
Urban (*N* = 27)	10,962 ± 15,104		2.43 ± 2.39	
Rural (*N* = 233)	7996 ± 9425		3.23 ± 3.72	
Types of cancer treatment		0.00		0.26
Life-extending treatment (*N* = 138)	10,808 ± 12,248		3.36 ± 3.97	
Conservative treatment (*N* = 122)	5201 ± 5584		2.83 ± 2.97	
Days from being diagnosed with cancer to dying	5549 ± 6033	0.00		0.00
≤3 months(*N* = 25)			2.50 ± 2.31	
3 months–6 months(*N* = 51)	4499 ± 4773		2.62 ± 2.71	
6 months-1 year(*N* = 58)	5130 ± 5306		2.70 ± 2.46	
1 year-2 years(*N* = 74)	7892 ± 8805		2.37 ± 2.05	
≥ 2 years(*N* = 52)	7474 ± 8201		5.43 ± 5.93	
Regions	14,638 ± 15,576	0.01		0.00
Western *N* = 53)	10,962 ± 15,104		2.34 ± 2.48	
Central (*N* = 106)	7996 ± 9425		4.81 ± 4.71	
Eastern (*N* = 101)			2.17 ± 2.08	

US$ Based on a currency exchange rate of the 6.6423 yuan to US$1.00 in 2016

## Discussion

This study revealed substantial urban-rural and regional disparities in various types of cancer treatments and cancer health care costs in the last three months of life of cancer patients between dying between June 2013 and June 2016 in China. Generally, urban patients and western region patients were significantly more likely to receive life-extending treatment, hospitalization and hospice care than those from rural areas or the eastern and western region. Previous studies of cancer patients showed that mean monthly health care costs increased as death approached [16] especially EOL hospital costs [20]. Our study revealed that about 40% of the health care expenses occur in the last three months of life, except for the western region with EOL costs just over 50% of total care costs. Patients who received life-extending treatment and inpatient care were more likely to incur higher EOL costs, which is consistent with previous studies that found most health care costs resulted from life-sustaining acute care in the last month of life [29, 44, 45]. Hospitalization tended to be the main driver of EOL health care costs in China, accounting for roughly 70% of EOL charges, which is consistent with research results in western countries [14–16, 24, 28].

The significant EOL differences in types of treatment, hospitalization and total care costs across regions, reflecting different regional levels of economic development, income and health care provision and social security provision [35, 46, 47]. The proportion hospitalized, the average per capita hospital admissions, per capita hospital days and medical expenditures were higher in the eastern region than that in the central region. Since medical resources were concentrated in the economically developed eastern region in China, these results were consistent with the difference between eastern and central region. Surprisingly, we found that health care utilization and health care expenditures were highest in western region. There are three possible reasons to explain this result. The cancer patients in western regions were more likely to be minorities and younger, than eastern patients, which is consistent with the previous studies showing that younger age was associated with higher medical costs in cancer patients [24]. Also, in the western region there were more married, and fewer widowed, patients than in the eastern region,and spouses may have been more likely to choose treatments to extend their partner’s life than non-partner carers [48]. In addition, the western region had many observations from Shaanxi and Yunnan provinces, with higher GDP and health provision than in other western region provinces,,such as Xingjiang,Inner Mongolia and Qinghai, which may have biased upwards the western region results. Finally, different sociocultural systems across regions, with different commitments to family care for cancer patients, likely impacted on treatment types and hospitalization rates [46, 48].

Although reimbursement of basic medical insurance helped reduce some of the economic burden imposed by health care expenses, we found that cancer patients still needed to self-pay about half their total EOL health care costs. Urban cancer patients had higher incomes, and their social networks were likely to have higher incomes, than rural cancer patients, which allowed them to access life-extending treatments and hospitalization costs more easily. Also, urban cancer patients had a higher capacity to pay-back borrowed funds than rural patients. We posit that the more limited capacity to borrow by rural cancer patients may have constrained their choice of treatment regime and hospital care. High borrowing requirements for central region patients may also have similarly restricted their treatment regime.

However, the lower EOL health care expenditure for rural cancer patients, and patients from the central and eastern regions, does not mean a more efficient use of medical resources. Rather, it points to the inequities in access to medical services, differences in incomes and disparities in health facilities, which constrained urban-rural and regional patients’ choice of treatment [14]. As shown in Table 1, most urban patients accessed municipal level or above hospitals, while village clinics were the nearest health facility for many rural patients. Over 50% of Western region patients accessed county hospitals and municipal or above hospitals, while more central region patients accessed private clinics and more eastern region patients accessed village health facilities.

The costs of EOL cancer care has increased dramatically [49], imposing increased burdens on China’s health care system. American studies reported that about 25% of all United States Medicare spending was for 5% patients who were in the last year of life [11]. Our study found that about half of the total cancer health care costs in the last three months of cancer patients’ life were covered by medical insurance. While some form of medical insurance is nearly universal in China [50], basic medical insurance did not cover all cancer treatments. Patients were forced to pay out-of-pocket expenses (including borrowing from family and friends) or go without treatment [4]. The out-of-pocket expenses accounted for about half of the total care costs, imposing a heavy economic burden on patients and their families.

In China, a hospital deposit is an out-of-pocket-expense required when patients are admitted to hospital, a proportion of which may be returned after cost settlement within 3–5 business days of discharge. We found that about one third of inpatients need to borrow money from others to cover inpatient fees. We recommend that policies and measures should be implemented by the national health insurance agency to help reduce the health care cost disparities between urban and rural locations and across regions. Further, clear clinical guidelines for the EOL care [14] and good EOL conversations between patients and their health care professionals [44] would improve EOL cancer patients’ decisions about health care options, especially for rural patients and patients from poor regions.

The use of hospital services for EOL care for cancer patients was higher in China than in the Canada [51] and the United States [15] where palliative care was more widespread. Palliative care was relatively underutilized in China, and EOL cancer patients received much less professional hospice services by medical staff than cancer patients in the United States [14, 15, 52] or England [22]. Since palliative cancer care services were associated with lower expenditures than hospital-based care [40], China needs to invest in palliative care facilities to improve the quality of life of cancer patients and their families and avoid over-treatment, which will attenuate medical costs and improve the quality of end-of-life care. Access to palliative care varied by urban-rural location and province. Consistent with previous studies in United States [14, 15], rural cancer patients in China were less likely to choose hospice care and to use inpatient hospital care at the end of life. There are several reasons for the under-utilization of palliative care. There are relatively few palliative care facilities. Since hospice care is still at the early development stage in China [28], patients (and many health professionals) lack an awareness of palliative care options. We recommend strengthening through education and publicity activities the benefits of hospice services to improve patients, doctors and health administrators’ awareness of the availability and benefits of hospice care.

Different from the “patient-oriented” culture in western countries, there is a“family-oriented” culture in China. In Chinese traditional culture, family members play an important role in making decisions about treatments to extend cancer patients’ lives [48]. Improving patients and carers’ knowledge about treatment options, especially hospice care, may decrease the use of hospital facilities [22]. However, cultural taboos about terminal illness and death are barriers to communication at the end of life [53]. Therefore, there is a lack of EOL conversations between patients and their professional doctors to guide appropriate cancer treatment decision-making at the end of life [44]. This requires better medical training and clear protocols to inform cancer patients and their families about treatment options.

Finally, we recommend a review of relevant government policies and practices to both provide both more palliative care and better information to cancer patients to choose appropriate cancer treatments. For individuals, it would be helpful to improve the quality of life during end -of-life care, lower medical costs and ease the financial burden of medical expenses for patients and their families. A better mix of health treatments and service facilities, such as palliative care, would ensure not only a more equitable access to health care for rural cancer patients, and those from disadvantages provinces, but a more efficient allocation of health resources, such as between hospital and hospice care.

We acknowledge a number of limitations in this study. First, we obtained the output variable by retrospective data reported by the family caregivers, raising the possibility of recall bias. Although we tried several methods to reduce the recall bias on the payments, we acknowledge that some data of payments might be missing or inaccurate. But, the health cost expenditure was a significant call on family resources and had to be paid before reimbursement claims from insurance provides confidence on the data. The average per capita health care expenditure was about $19,758 and the average out-of-pocket expenditure is about $11,287 in our study. This compares favorably with the average per capita health care expenditure was about $22,582 for cancer patients from a patients in Jilin province [54] and a RDPDC report that the average per capita out-of-pocket expenditure was about $9900 for lung and stomach cancer and about $10,000 for Colorectal cancer and esophageal cancer [55].

Second, although the study examined the total health care costs in the last three months of life, we did not break down the costs of hospice care, drugs, and other treatment types. Future work needs to calculate the cost differential between EOL hospice expenditures versus hospital costs. Third, we started to explore regional differences across three broad geographical regions. This preliminary work revealed the need for more detailed regional investigations of cancer costs and treatments, adjusting for level of development, medical expenditures, types of treatment and health care facilities.

Finally, our conclusions based on the snowball sampling might be biased. Snowball sampling is a non-probabilistic sampling technique, where the elements are not randomly drawn, but are dependent on the subjective choices of respondents. We might have oversampled a particular social network of peers, if the sample included an over-representation of individuals with social connections who share similar characteristics [56]. While we implemented various measures to attenuate any selection bias, we call for further studies of deceased cancer patients in other parts of China. These limitations need to be taken into consideration in further studies.

## Conclusion

EOL cancer care costs imposed a heavy economic burden on China’s health system and on family members and friends of cancer patients. Between rural and urban cancer patients, and between cancer patients from different regions, we revealed significant disparities in types of cancer treatment, utilization of medical care facilities and health care expenditures in the last three months of life in China, including differences. Related medicare policies, reasonable clinical guidelines and good EOL conversations among patients, family caregivers and doctors are essential to guiding patients to use appropriate EOL treatments, access medical facilities and manage cancer care expenditures. These recommendations will attenuate EOL cancer care costs, reduce treatment differentials, increase health care facility equity and access and narrow health outcomes between cancer patients from different urban-rural locations and regions.

## Additional files

Additional file 1: Questionnaire for health care utilization, health care expenditure and treatment decisions at End-of-life. (DOC 54 kb)

Additional file 2: A: Distributions of health care costs of cancer patients from being diagnosed with cancer to dying by rural-urban differences (*N* = 792). B: Distributions of health care costs of cancer patients from being diagnosed with cancer to dying by geographical location (*N* = 792). (DOC 38 kb)

Additional file 3: A: Distributions of health care costs of cancer patients at the last three months of life from rural-urban differences (*N* = 792). B: Distributions of health care costs of cancer patients at the last three months of life from different treatment (*N* = 792). C: Distributions of health care costs of cancer patients at the last three months of life by geographical location (*N* = 792). (DOCX 21 kb)

## Abbreviations

**EOL**: End of life
**GLMs**: Generalized linear models
**ICD-10**: the International Statistical Classification of Diseases and Related Health Problems, Tenth Revision
**SS**: Snowball sampling
**US$**: Unite State Dollars
