# Supplementary material for: Geographical disparities in treatment and health care costs for end-of-life cancer patients in China: a retrospective study
Source: BMC Cancer. 2019 Jan 8;19:39. doi: 10.1186/s12885-018-5237-1 (PMC6325809; doi:10.1186/s12885-018-5237-1)
Supplement: Supplementary file 1 — Questionnaire for health care utilization, health care expenditure and treatment decisions at End-of-life. (DOC 54 kb) [file 12885_2018_5237_MOESM1_ESM.doc]

**Questionnaire for health care utilization, health care expenditure and treatment decisions at End-of-life**

【Introduction】Hi! We are researchers from XXX University. We are now conducting a scientific research project on a survey about medical service and payment at end-of-life. Please answer all the questions below carefully and fill in a number in the blank according to your actually personal situation. We will strictly abide by Chinese Statistic Law and be strict confidentiality of the information you provide, Thank you for your support and cooperation!

**Section II. Patient Information**

1. Gender：_____ ①Male ②Female

2. Age:_____

3. The date of death : (Gregorian calendar /Lunar calendar) ______Year _____Month ___ _Day

4. The date of birth : (Gregorian calendar /Lunar calendar) ______Year _____Month ___ _Day

5. Where is the place that patient passed away?

①Home ②Hospital ③the way to hospital ④Nursing home ⑤Hospice institution ⑥Other(Please specify)____

6.Marital status:

①Widowed ②Married ③Unmarried ④Divorced

7.The nation to which the end-of-life patient belongs:

①Han ②Other______

8. The job of the end-of-life patient:

①Farmer ②Migrant workers ③Enterprise employees ④Individual business ⑤Teacher ⑥Doctor ⑦Village cadres

⑧Soldier ⑨Civil service ⑩Retirement (The job before he/she retired ______) ⑪Other (Please specify)____

9. Which is the type of medical insurance that patient took in?  (Multiple choice)

①New Rural Cooperative Medical Care (NRCMS) ②Urban Employee Basic Medical Insurance (UEBMI)

③Urban Resident Basic Medical Insurance (URBMI) ④Commercial insurance ⑤Public Medicare ⑥Without insurance

⑦Other(Please specify)____

10. Annual household income : _______ ￥(RMB)

11. Residential address: __________Province________City _________County

12. Place of residence: ①Urban area ②Urban-rural fringe area ③Rural area

13. The nearest medical institution to patient’s house (or apartment): _________(name) ; And the distance between the nearest medical institution and patient’s house (apartment) : _________Kilometers (km)

14. Who lived with patient at the three months of life ? (Multiple choice)

①Living alone ②Spouse ③Children ④Parents ⑤Grand children ⑥Grand parents ⑦Carers ⑧Friends ⑨Others (Please specify)____

15. What kind of cancer did patient suffer from? ___________

①Lung cancer ②Colon cancer ③Breast cancer ④Gastric cancer ⑤Hepatoma ⑥Renal cancer ⑦Rectum cancer

⑧Esophagus cancer ⑨Bladder cancer ⑩Cervical cancer ⑪Malignant lymphoma ⑫Prostate cancer ⑬Endometrial cancer ⑭Ovarian cancer ⑮Thyroid cancer ⑯Other(Please specify)____

**Section III. Health care utilization and health care expenditure of patient**

1. From being diagnosed with cancer to death:

(1) How many days did the patient survive ? days.

1. The total medical costs were: ￥(RMB), of which out-of-pocket expenses were : ￥(RMB),

the reimbursements were: ￥(RMB), and nursing costs and delay compensation were  ￥(RMB).

1. In the last three months of life:
2. The total medical costs were: ￥(RMB), of which out-of-pocket expenses were : ￥(RMB),

the reimbursements were: ￥(RMB), and nursing costs and delay compensation were ￥(RMB).

1. In the last three months of life:
2. Did the patient receive inpatient service? ①Yes ②No ***（If it didn’t happen, please skip to question19）***
3. The total number of hospital admissions:
4. How many are hospitalization days？________days.

(4)The total medical costs of hospitalization were: ￥(RMB), of which out-of-pocket expenses were : ￥(RMB),

the reimbursements were: ￥(RMB), and nursing costs and delay compensation were ￥(RMB).

19.Whether medical specialists provide professional hospice care？（Whether a medical institution hospice care services palliative medicine department intervened？）

① Yes ② No ***(skip to question21)*** ③ Uncertain ***(skip to question21)***

20(1) Please write down the name of the medical institution which provide professional hospice care, _________________,

and what type of medical institution? ______

① Village health care station ② Community health care center ③ County and above level hospitals ④ Tumour hospital

⑤ Private hospital ⑥ Other reasons(Please specify)___ _

20(2) Where was the hospice care service provided?

① Completely at the medical institution ② Completely at patient’s home ③ At the medical institution and patient’s home

④ Other (Please specify)___ _

**Section IV: Treatment decisions at End-of-life**

21. What kind of treatment did the **patient** received after the **doctor** first confirmed that the continued treatment didn’t work?

① Choosing life-extending treatment ② Choosing conservative treatment

22.To what extent do you think the medical costs of treatment for the last three months of life impose a financial burden on the family?

① Not at all ② A little ③Moderately ④ Very heavy ⑤ Extremely

23.To what extent do you think the medical costs of treatment throughout the cancer treatment impose a financial burden on the family?

① Not at all ② A little ③Moderately ④ Very heavy ⑤ Extremely

24.Did patient or family member borrow any money from relatives, friends or colleagues for the medical treatment?

① Yes ② No ***(skip to question26)***

25(1) How much were the borrowed money? ________ ￥(RMB),

25(2) How long can patient or family member pay off the loan? ________ (years)

26. Are you satisfied with the medical services the patient received in the last three months of life?

① Very dissatisfied ② A little dissatisfied ③ Moderately ④ A little satisfied ⑤ Very satisfied

THE END
