# Supplementary material for: Geographical disparities in treatment and health care costs for end-of-life cancer patients in China: a retrospective study
Source: BMC Cancer. 2019 Jan 8;19:39. doi: 10.1186/s12885-018-5237-1 (PMC6325809; doi:10.1186/s12885-018-5237-1)
Supplement: Supplementary file 2 — A: Distributions of health care costs of cancer patients from being diagnosed with cancer to dying by rural-urban differences (N = 792). B: Distributions of health care costs of cancer patients from being diagnosed with cancer to dying by geographical location (N = 792). (DOC 38 kb) [file 12885_2018_5237_MOESM2_ESM.doc]

Additional File 1

A: Distributions of health care costs of cancer patients from being diagnosed with cancer to dying by rural-urban differences (N=792)

| Characteristics | Urban (N=195) | | | Rural (N=597) | | | P-value |
| --- | --- | --- | --- | --- | --- | --- | --- |
|  | Mean (SD) | Median | 25%-75% | Mean (SD) | Median | 25%-75% |  |
| Per capita expenditures, US$ | 32,671  (45,576) | 22,582 | 12,044-34,325 | 15,541  (15,955) | 10,237 | 5,269-21,077 | 0.00 |
| Including:out-of-pocket expenses | 17,051  (23,731) | 10,539 | 4,517-18,066 | 9,405  (10,623) | 6,022 | 3,011-12,044 | 0.00 |
| Reimbursements | 15,620  (29,698) | 9,033 | 4,517-18,066 | 6,136  (8,202) | 3,011 | 1,204-7,527 | 0.00 |
| US$ Based on a currency exchange rate of the 6.6423 yuan to US$1.00 in 2016. | | | | | | | |

B: Distributions of health care costs of cancer patients from being diagnosed with cancer to dying by geographical location (N=792)

| Characteristics | Western (N=162) | | | Central (N=242) | | | Eastern (N=388) | | | P-value |
| --- | --- | --- | --- | --- | --- | --- | --- | --- | --- | --- |
|  | Mean (SD) | Median | 25%-75% | Mean (SD) | Median | 25%-75% | Mean (SD) | Median | 25%-75% |  |
| Per capita expenditures,US$a | 19,791  (18,132) | 15,055 | 8,431-27,165 | 16,579  (20,423) | 9,033 | 4,818-21,077 | 21,703  (33,747) | 13,550 | 6,549-27,099 | 0.00 |
| Including:out-of-pocket expenses | 8,979  (11,036) | 5,645 | 3,011-11,291 | 10,940  (15,516) | 6,022 | 2,559-13,550 | 12,483  (16,579) | 7,528 | 4,185-15,055 | 0.04 |
| Reimbursements | 10,812  (12,365) | 7,528 | 2,710-14,076 | 5,639  (8,642) | 3,011 | 1,506-6,022 | 9,220  (21,479) | 45,17 | 1,506-10,539 | 0.00 |
| US$ Based on a currency exchange rate of the 6.6423 yuan to US$1.00 in 2016. | | | | | | | | | | |
