# Supplementary material for: Geographical disparities in treatment and health care costs for end-of-life cancer patients in China: a retrospective study
Source: BMC Cancer. 2019 Jan 8;19:39. doi: 10.1186/s12885-018-5237-1 (PMC6325809; doi:10.1186/s12885-018-5237-1)
Supplement: Supplementary file 3 — A: Distributions of health care costs of cancer patients at the last three months of life from rural-urban differences (N = 792). B: Distributions of health care costs of cancer patients at the last three months of life from different treatment (N = 792). C: Distributions of health care costs of cancer patients at the last three months of life by geographical location (N = 792). (DOCX 21 kb) [file 12885_2018_5237_MOESM3_ESM.docx]

Additional File 2

A: Distributions of health care costs of cancer patients at the last three months of life from rural-urban differences (N=792)

| Characteristics | Urban (N=195) | | | Rural (N=597) | | | P-value |
| --- | --- | --- | --- | --- | --- | --- | --- |
|  | Mean (SD) | Median | 25%-75% | Mean (SD) | Median | 25%-75% |  |
| Health expenditures |  |  |  |  |  |  |  |
| Per capita expenditures,US$ | 12,501 (15,711) | 10,184 | 5,969-14,701 | 5,996 (6,359) | 4,184 | 1,925-9,001 | 0.00 |
| Including:out-of-pocket | 6,225 (7,221) | 4,516 | 1,957-7,828 | 3,893 (4,693) | 2,421 | 1,204-4,640 | 0.00 |
| reimbursement | 6,276 (11,558) | 3,763 | 1,506-6,323 | 2,103 (2,972) | 903 | 0-3,011 | 0.00 |
| Inpatient health care utilization |  |  |  |  |  |  |  |
| Per capita hospital expenditures, US$ | 10,085 (12,384) | 7,527 | 3,011-11,893 | 4,320 (5,798) | 2,710 | 0-6,022 | 0.00 |
| Including:out-of-pocket | 4,668 (5,696) | 3,010 | 1,355-6,020 | 2,570 (4,241) | 1,500 | 0-3,011 | 0.00 |
| reimbursement | 5,417 (9,025) | 3,011 | 1,355-6,022 | 1,750 (2,650) | 753 | 0-2,258 | 00.00 |
| US$ Based on a currency exchange rate of the 6.6423 yuan to US$1.00 in 2016. | | | | | | | |

B: Distributions of health care costs of cancer patients at the last three months of life from different treatment (N=792)

| Characteristics | Life-extending treatment (N=379) | | | Conservative treatment (N=413) | | | P-value |
| --- | --- | --- | --- | --- | --- | --- | --- |
|  | Mean (SD) | Median | 25%-75% | Mean (SD) | Median | 25%-75% |  |
| Health expenditures |  |  |  |  |  |  |  |
| Per capita expenditures,US$ | 10,601 (12,611) | 8,679 | 4,334-13,970 | 4,841(5,550) | 3,431 | 1,926-6,593 | 0.00 |
| Including:out-of-pocket | 5,852 (6,574) | 3,914 | 1,807-7,527 | 3,196 (3,914) | 2,002 | 1,053-3,914 | 0.00 |
| reimbursement | 4,749 (8,768) | 3,011 | 752.75-5,570 | 1,645 (2,596) | 752 | 0-2,258 | 0.00 |
| Inpatient health care utilization |  |  |  |  |  |  |  |
| Per capita hospital expenditures, US$ | 8,467 (10,397) | 6,022 | 2,409-11,743 | 3,236 (4,516) | 1,807 | 0-4,516 | 0.00 |
| Including:out-of-pocket | 4,409 (5,738) | 3,010 | 1,053-6,020 | 1,873 (3,094) | 903 | 0-2,560 | 0.00 |
| reimbursement | 4,058 (6,977) | 2,258 | 530-4,516 | 1,363 (2,275) | 452 | 0-1,807 | 0.00 |
| US$ Based on a currency exchange rate of the 6.6423 yuan to US$1.00 in 2016. | | | | | | | |

C: Distributions of health care costs of cancer patients at the last three months of life by geographical location (N=792)

| Characteristics | Western (N=162) | | | Central (N=242) | | | Eastern (N=388) | | | P-value |
| --- | --- | --- | --- | --- | --- | --- | --- | --- | --- | --- |
|  | Mean (SD) | Median | 25%-75% | Mean (SD) | Median | 25%-75% | Mean (SD) | Median | 25%-75% |  |
| Health expenditures |  |  |  |  |  |  |  |  |  |  |
| Per capita expenditures,US$ | 9,808 (8.910) | 8,679 | 3,431-13,578 | 5,814  (6,372) | 4,184 | 1,925-14,722 | 7,756 (11,948) | 5,668 | 2,678-10,206 | 0.00 |
| Including:out-of-pocket | 5,063 (5,917) | 3,613 | 1,536-6,533 | 3,976 (4,656) | 2,559 | 1,204-4,667 | 4,516 (5,802) | 3,011 | 1,500-6,022 | 0.00 |
| reimbursement | 4,745 (5,301) | 3,011 | 867-7,527 | 1,838 (2,772) | 903 | 0-2,258 | 3,240 (8,270) | 1,500 | 0-4,291 | 0.00 |
| Inpatient health care utilization |  |  |  |  |  |  |  |  |  |  |
| Per capita hospital expenditures, US$ | 8,225 (8,637) | 6,022 | 2,258-12,044 | 4,086 (5,740) | 2,409 | 0-5,720 | 5,700 (9,223) | 3,011 | 435-7,528 | 0.00 |
| Including:out-of-pocket | 3,978 (5,773) | 2,635 | 903-4,517 | 2,490 (4,045) | 1,500 | 0-3,010 | 3,075 (4,598) | 1,505 | 166-3,839 | 0.00 |
| reimbursement | 4,247 (5,127) | 2,258 | 0-6,323 | 1,596 (2,489) | 752 | 0-1,806 | 2,625 (6,327) | 994 | 0-3,000 | 0.00 |
| US$ Based on a currency exchange rate of the 6.6423 yuan to US$1.00 in 2016. | | | | | | | | | | |
